# Odontogenic ameloblast-associated protein (ODAM) inhibits growth and migration of human melanoma cells and elicits PTEN elevation and inactivation of PI3K/AKT signaling

**DOI:** 10.1186/1471-2407-13-227

**Published:** 2013-05-07

**Authors:** James S Foster, Lindsay M Fish, Jonathan E Phipps, Charles T Bruker, James M Lewis, John L Bell, Alan Solomon, Daniel P Kestler

**Affiliations:** 1 Department of Medicine, Human Immunology and Cancer Program, University of Tennessee Health Sciences Center-Knoxville, 1924 Alcoa Highway, Knoxville, TN, 37920, USA; 2 Department of Surgery, Surgical Oncology and Cancer Institute, University of Tennessee Health Sciences Center-Knoxville, 1924 Alcoa Highway, Knoxville, TN, 37920, USA; 3 Graduate School of Medicine, University of Tennessee Health Sciences Center-Knoxville, 1924 Alcoa Highway, Knoxville, TN, 37920, USA; 4 Department of Pathology, Boca Raton Regional Hospital, 800 Meadows Road, Boca Raton, FL, 33486, USA

## Abstract

**Background:**

The Odontogenic Ameloblast-associated Protein (ODAM) is expressed in a wide range of normal epithelial, and neoplastic tissues, and we have posited that ODAM serves as a novel prognostic biomarker for breast cancer and melanoma. Transfection of ODAM into breast cancer cells yields suppression of cellular growth, motility, and in vivo tumorigenicity. Herein we have extended these studies to the effects of ODAM on cultured melanoma cell lines.

**Methods:**

The A375 and C8161 melanoma cell lines were stably transfected with ODAM and assayed for properties associated with tumorigenicity including cell growth, motility, and extracellular matrix adhesion. In addition, ODAM–transfected cells were assayed for signal transduction via AKT which promotes cell proliferation and survival in many neoplasms.

**Results:**

ODAM expression in A375 and C8161 cells strongly inhibited cell growth and motility *in vitro*, increased cell adhesion to extracellular matrix, and yielded significant cytoskeletal/morphologic rearrangement. Furthermore, AKT activity was downregulated by ODAM expression while an increase was noted in expression of the PTEN (phosphatase and tensin homolog on chromosome 10) tumor suppressor gene, an antagonist of AKT activation. Increased PTEN in ODAM-expressing cells was associated with increases in PTEN mRNA levels and *de novo* protein synthesis. Silencing of PTEN expression yielded recovery of AKT activity in ODAM-expressing melanoma cells. Similar PTEN elevation and inhibition of AKT by ODAM was observed in MDA-MB-231 breast cancer cells while ODAM expression had no effect in PTEN-deficient BT-549 breast cancer cells.

**Conclusions:**

The apparent anti-neoplastic effects of ODAM in cultured melanoma and breast cancer cells are associated with increased PTEN expression, and suppression of AKT activity. This association should serve to clarify the clinical import of ODAM expression and any role it may serve as an indicator of tumor behavior.

## Background

Melanoma is the most lethal form of skin cancer and the incidence is increasing in the United States and worldwide [1]. Mortality from melanoma occurs as a result of local tumor proliferation and invasion of surrounding tissues leading to metastatic spread of the disease. Clinically, metastases are often predicted by primary tumor factors that reflect biologic behavior such as Breslow thickness, mitotic rate, and ulceration. Sentinel lymph node (SLN) status remains the single most important predictor of survival [2]. Recently, multiple potential biomarkers for melanoma have been identified; however, their clinical significance remains largely to be determined [3-5]. On a molecular and genetic level, a number of factors influencing primary melanoma growth and metastasis have been identified, including signaling via the phosphoinositide 3-kinase (PI3K)/AKT/mammalian target of rapamycin (mTOR), and Wnt/β-catenin pathways, as well as BRAF mutations which activate signaling through the Ras/Raf/MAP-ERK kinase (MEK)/mitogen-activated protein kinase (/MAPK) pathway [6-9].

The Odontogenic Ameloblast-Associated Protein (ODAM) was first identified less than a decade ago as the protein constituent of calcifying epithelial odontogenic/Pindborg tumors (CEOT) and subsequent studies revealed that it is highly expressed in mature ameloblasts and present in the rodent enamel organ and junctional epithelium [10-13]. It has also been found to be present in additional normal human tissues including the skin, gastrointestinal tract, trachea, bronchus, and glandular breast epithelium. Further analysis showed that ODAM is also expressed in epithelial malignancies including those of the, colon, breast, lung, stomach, and in melanoma [14-16]. In breast cancer patient biopsies a correlation was observed between ODAM expression/localization and disease staging/clinical outcome, indicating that ODAM may serve as a novel prognostic biomarker in this type of cancer [17]. When stably transfected with recombinant ODAM the MDA-MB-231 breast cancer cell line showed marked inhibition of neoplastic and metastatic properties *in vivo* and *in vitro*[18]. This suggests that ODAM has a potentially significant role in regulating tumorigenesis and metastasis in breast cancer with possible clinical implications. More recently, a retrospective study of melanoma patient samples have demonstrated a significant correlation of ODAM expression/nuclear localization and sentinel lymph node metastases indicative of poorer prognosis [19].

The apparent association of ODAM expression with disease status in breast cancer and melanoma, and the inhibition of neoplastic and metastatic properties shown in ODAM-transfected breast tumor cells have led us to investigate the role of this protein in the tumorigenesis of melanoma. To this end the invasive C8161 and A375 human melanoma cell lines were stably transfected with a construct encoding ODAM and evaluated *in vitro* for properties associated with tumorigenesis. Similar to our earlier studies with breast cancer cells, the results indicate that ODAM expression inhibits cell growth and migration in melanoma cells. We further demonstrate that this inhibition is associated with increased expression of the PTEN (phosphatase and tensin homolog on chromosome 10) tumor suppressor and suppression of signaling via AKT, in both of the melanoma cell lines as well as in MDA-MB-231 breast cancer cells.

## Methods

### Cells and tissue culture

The human melanoma cell line C8161 [20] was kindly provided by Professor Mary JC Hendrix. The A375 melanoma cell line and BT-549 breast cancer line were obtained from the American Type Culture Collection (Rockville, MD). Control and ODAM-expressing MDA-MB-231 cells were described in detail previously [18]. All cell cultures were maintained in DMEM/F12 medium (Lonza, Walkersville, MD) containing 5% fetal bovine serum (FBS, Thermo-Fisher-Hyclone, Logan, UT), and penicillin/streptomycin (Thermo-Fisher, Pittsburg, PA) in a humidified incubator at 37°C under 5% CO_2_. These studies did not involve human or animal subjects but all studies were carried out under the oversight of our Institutional Review Board (approval numbers 2683 and 2803), Biosafety Commitee (approval numbers 251-11 and 334-11), and Animal Care and Use Commitee (approval number 2092-0412).

### Transfection of tumor cell lines with rODAM

The C8161, A375, and BT-549 cell lines were transfected with either a human ODAM-pcDNA5T/O construct [18] or, the empty vector control using Lipofectamine LTX reagent (Invitrogen, Carlsbad, CA) according to the manufacturer’s protocol. Selection of stable ODAM-producing clones was performed in medium supplemented with 400 μg/mL hygromycin (Thermo-Fisher-Hyclone) in 100-mm culture dishes and visible colonies transferred into 24-well plates. Culture media collected 7–10 days later were tested for ODAM production by capture ELISA [18]. ODAM-positive clones were designated as C8161-ODAM, A375-ODAM, BT-549-ODAM, and along with respective controls were expanded and maintained in medium with hygromycin.

### Cell growth assays

Control and ODAM-expressing clones of A375, C8161, and BT-549 cells were trypsinized, counted, and plated in quadruplicate in 12-well plates at 1×10^4^ cells/well with standard growth medium. At appropriate intervals, cells were fixed by addition of 70% ethanol and stained with 0.1% crystal violet. After washing with water, the crystal violet was solubilized with 10% acetic acid and the relative cell content measured as absorbance at 562 nm. Where applicable, growth rates were determined by linear regression analysis using GraphPad Prism 4.0 software.

### Cell migration assays

Trypsinized control and ODAM-expressing melanoma cell lines were washed and suspended (5×10^5^ cells/mL) in serum-free DMEM/F12 medium and a 100 μL aliquots were placed in the upper chamber of a Costar Transwell permeable support (8-μm pore size, Thermo-Fisher); the lower chamber was filled with 0.6 mL of DMEM/F12 medium with 10% FBS serving as a chemo-attractant. After incubation at 37&z.ousco;C for 18 h, the membrane was fixed and stained with HEMA3 Wright-Giemsa (Thermo-Fisher). Non-migrating cells were swabbed from the upper surface and those that passed through to the lower surface were photographed with an inverted microscope and counted.

### Immunofluorescent/Cytoskeletal staining

Control and ODAM-expressing cells were plated onto 15-mm sterile glass coverslips (Thermo-Fisher) in 12-well tissue culture plates (BD Biosciences, San Jose, CA) and, 72 h later, washed with PBS, fixed with 4% paraformaldehyde, permeabilized with 0.25% Triton X-100/PBS, and blocked with 4% goat serum in PBS. Cellular F-actin was visualized by staining with AlexaFluor488-conjugated Phalloidin (Invitrogen) and Hoescht 33342 nuclear counterstain (Roche Applied Science, Indianapolis, IN). ß-catenin was visualized on separate slides by staining with rabbit anti-ß-catenin (Thermo-Fisher-Neomarkers, Fremont, CA) followed by AlexFluor 488-conjugated goat anti-rabbit IgG (Invitrogen) along with Hoescht 33342. For confocal/SIM microscopy images were collected on a Zeiss LSM 710 confocal laser scanning microscope equipped with 405 nm and 488 nm laser lines using a Plan-Apochromat 40×/1.4 oil objective (Carl Zeiss Microimaging, Thornwood, NY). Where applicable optical sections were collected at 1 μm spacing and shown as maximum intensity projections using Zen 2009 software (Carl Zeiss).

### Western blot analysis

For Western blot analysis [21], cells growing at ~80% confluence in 100 mm dishes were washed in cold PBS and lysed in RIPA buffer (20 mM Tris, pH 7.5, 200 mM NaCl, 0.5% Triton X-100, 0.2% sodium deoxycholate, 0.15% SDS, 1mM sodium orthovanadate, 5 mM sodium fluoride, 5 mM β-glycerophosphate and 0.5 mM PMSF) followed by centrifugation at 15,000 × g for 20 min at 4°C. Lysate protein concentrations were determined by BCA protein assay (Thermo-Fisher-Pierce, Rockwood, IL) and equal 50-100 μg amounts (control vs. ODAM-expressing cultures) were electrophoresed in 10% Bis-Tris gels (Invitrogen) and blotted to PVDF membranes. Equal protein loading was verified by Ponceau S staining and by reprobing blots for β-actin expression. For detection of ODAM production cell supernatants (1 ml) were subjected to immunoprecipitation with anti-ODAM monoclonal antibody 8B4 as described, blotted, and probed with anti-ODAM antibody 5A1 [15,18,21]. Additional primary antibodies used were rabbit monoclonal anti-PTEN (D4.3)XP, rabbit anti-phospho-AKT (Ser 473), anti-phospho-AKT (Thr 308), anti-total AKT, anti-phosph-PDK1, anti-phospho-PI3Kp85 (Y458)/p55 (Y199), and anti-phospho-c-Raf (S259) (all from Cell Signaling Technologies, Danvers, MA); anti-phospho-Erk (sc-7383), anti-Erk2 (sc-154), anti-PI3K (sc-423), and anti-Erk1 (SC-93) (all from Santa Cruz Biotech, Santa Cruz, CA). Anti-β-actin was from Sigma-Aldrich (St. Louis, MO). Polyclonal rabbit anti-PTEN (Ab-2) was from Neomarkers (Freemont, CA). Anti-ODAM monoclonal antibodies 5A1 and 8B4 are produced in our laboratory. Probed blots were developed using HRP-conjugated secondary antibodies (Jackson Immunoresearch, Westgrove, PA) with chemiluminescent substrate detection (ECL, Thermo-Fisher-Pierce) visualized on Kodak X-OMAT LS film. For probing with multiple antibodies lysates were run on replicate gels or blots were reprobed after stripping with 1% SDS in 50 mM glycine, pH 3.0 [22].

### Cell-substrate adhesion assays

Polystyrene 96-well tissue culture plates were coated overnight at 4°C with 50 μL/well of Matrigel (BD Biosciences) or BSA, each at a concentration of 50 μg/mL. After washing with PBS, the wells were filled with 50 μL of suspended, trypsinized cells (5×10^5^ cells/mL) and the plates incubated at 37°C for 40 minutes. After washing with PBS, the cells were fixed for 30 min with 4% glutaraldehyde and washed with water. The relative cell binding was determined after staining with 0.1% crystal violet, solubilization with 10% acetic acid, and measurement of absorbance at 562 nm [18].

### RNA isolation and analysis by real time RT-PCR

Total cellular RNA was harvested from control and ODAM-expressing melanoma cultures by the RNAeasy Plus RNA isolation kit (Qiagen, Valencia, CA) and product integrity assessed by agarose gel electrophoresis. RNA concentration was determined by UV spectroscopy and first strand cDNA was synthesized using SuperScript III reverse transcriptase (Invitrogen) and 500 ng of RNA. Gene specific primers for PTEN were designed: (forward), 5´-TTTGAAGACCATAACCCACCAC-3´ and (reverse), 5´-ATTACACCAGTTCGTCCCTTTC-3´ (yielding a 134-bp product). Primers to human GAPDH (Real Time Primers, Elkins Park, PA) were used to amplify the calibrator gene: (forward), 5´-GAGTCAACGCGGATTTGGTCGT-3´ and (reverse), 5´-TTGATTTTGGAGGGATCTCG-3´ (yielding a 238-bp product). Real-time PCR was performed in 96-well PCR plates with an ICycler PCR unit (Bio-Rad, Hercules, CA) utilizing iQ SYBR Green Supermix containing 400 nM primer mix and 3 μl cDNA in a 20μl reaction volume. Fluorescence was detected with an iQ5 Multicolor Real-Time PCR system and analyzed with iQ5 optical systems software. Conditions for activation and denaturation were: cycle 1, 95°C for 3 min, followed by forty 30-sec amplification cycles at 95°C, 63°C, and 72°C.

### Metabolic labeling and immunoprecipitation

Control and ODAM-expressing A375 cells were pre-incubated in methionine/cysteine-free RPMI (MP Biomedicals, Santa Ana, CA) for 30 min. and labeled for 1 hour in the same medium containing 40 μCi/ml ^35^S TranS label (1175 Ci/mmol, MP Biomedicals, Irvine, CA). Cultures were then washed in PBS, lysed in RIPA buffer as above, and pre-cleared 4 hours with protein A/G agarose (Santa Cruz Biotechnology). Lysate amounts were equalized on the basis of trichloroacetic acid-precipitable counts, and PTEN was immunoprecipitated by incubation overnight with monoclonal rabbit anti-PTEN (Cell Signaling Technologies) and protein A/G agarose beads. The precipitates were centrifuged, washed in RIPA buffer, and proteins released by boiling in SDS sample buffer before separation by SDS-PAGE as above. Gels were soaked in 1M sodium salicylate (Sigma), dried, and exposed to Kodak X-OMAT LS film.

### Depletion of PTEN expression using siRNA

Control and ODAM-expressing melanoma cell lines were plated in 12-well plates at 30% confluency and transfected the following day with 40 pmol/well of PTEN siRNA (Cell Signaling Technologies) or a non-silencing control siRNA (Qiagen) using 2 μl/well Lipofectamine 2000 (Invitrogen) according to the manufacturers protocol. Following 72 hours in culture after transfection the cells were lysed for western blot analysis of PTEN expression and AKT phosphorylation as given above.

## Results

### Reduced growth and cellular migration as a result of ODAM-expression

**Figure 1:**
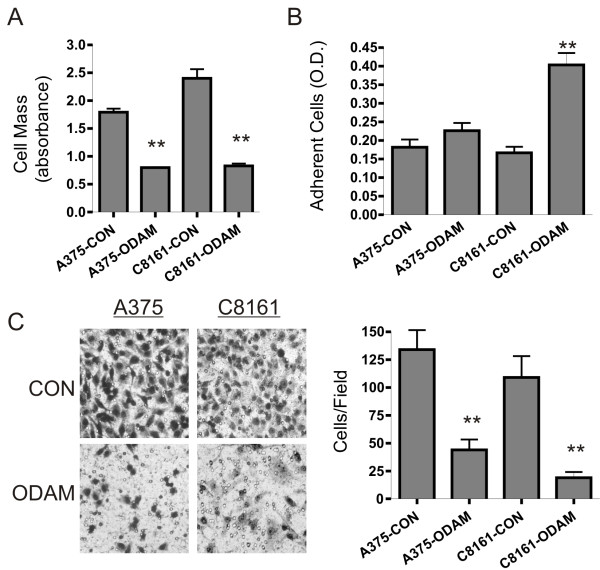
**Effect of ectopic ODAM expression on growth, adhesion, and migration of human melanoma cell lines. A**) Growth of control and stably ODAM-transfected A375 and C8161 melanoma cells as assessed by relative cell mass after six days of culture. Values are given as mean ± 1 standard deviation (S.D.) from quadruplicate cultures (**, p< 0.01). **B**) Adhesion of control and ODAM-expressing melanoma cell lines to matrigel-coated plastic surfaces. Values are based on absorbance of adherent cells and are given as mean ± 1 S.D. for six replicates (**, p< 0.01). **C**) Transwell migration assay of control and ODAM-expressing melanoma cell lines (left panels, Wright-Giemsa staining, original magnifications 200X). Average cell counts from nine representative fields for each determination are given as mean ± 1 S.D. (**, p< 0.01).

Prior studies with the MDA-MB-231 breast cancer cell line demonstrated that stable ODAM-expression suppressed the tumorigenic properties of these cells, as evidenced by reduced growth, cellular migration and barrier invasion *in vitro*, in addition to increased cellular adhesion, and an increased apoptotic rate [18]. Moreover, *in vivo* tumor growth was drastically reduced, as demonstrated by xenograft and metastatic models. Given the evidence that ODAM is expressed in melanoma and corresponds with lymph node metastasis [19], we wished to examine the effects of ODAM expression on melanoma cell lines. Initial experiments determined that the parental A375 and C8161 cell lines did not express detectable ODAM protein. After transfection, selection, and expansion, stable ODAM-expressing clones of these cell lines were characterized. As in previous studies [13,18] secreted ODAM was readily detectable in cell culture supernatants and was only associated with cells at low levels, primarily localized to the golgi apparatus (data not shown). *In vitro* growth assays revealed significant growth suppression in ODAM-expressing clones of both A375 and C8161 cells relative to controls after 6 days in culture, as shown by their differences in relative cell mass (Figure 1A). Similar decreased rates of growth in tissue culture were observed in additional ODAM-transfected clones of each cell line and were consistently observed upon routine cell passage.

In previous studies with MDA-MB-231 cells ODAM expression increased cell binding to extracellular matrix components and elicited direct cell-cell interactions in suspension [18]. Other investigators have observed ODAM localization at the tissue/enamel junctional epithelium where it is thought to act in part to promote cellular adhesion around the mature tooth [13]. Both A375-ODAM and C8161-ODAM cells exhibited increased adhesion on Matrigel-coated plates although the extent of this increase was greater in C8161 cells (Figure 1B). In contrast to our observations with MDA-MB-231 cells [18] neither melanoma cell line exhibited adhesive cell-cell interactions in suspension, regardless of ODAM expression.

Cellular migration, a critical component of tumor metastasis, is subject to complex regulation through cell adhesion to extracellular matrix components *in vitro* and in vivo [23]. Previously ODAM expression in MDA-MB-231 cells was shown to markedly inhibit cellular migration and barrier invasion [18]. Correspondingly, examination of the migratory abilities of the ODAM-expressing melanoma cell lines in transwell migration assays demonstrated that cell motility is strongly inhibited (70-80%) by ODAM expression in both A375 and C8161 melanoma cell lines (Figure 1C).

### Cytoskeletal rearrangement and cellular confirmation change

**Figure 2:**
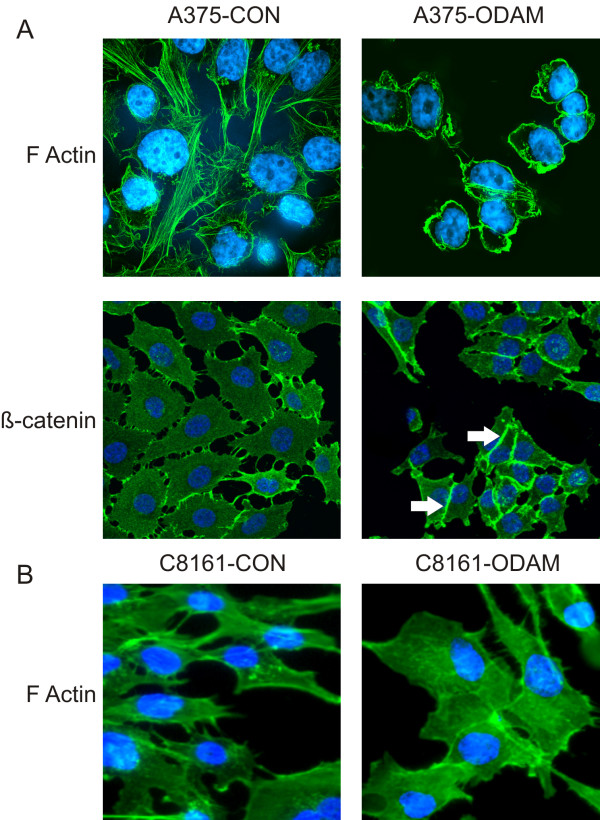
**Cytoskeletal rearrangement in ODAM-expressing human melanoma cell lines. A**) F-actin arrangement in A375-CON and A375-ODAM cells (top panels) was visualized by phalloidin staining (green) with nuclei counterstained (blue); original magnifications 320X). ß-catenin localization (lower panels) visualized by staining with anti-ß-catenin (green) with nuclei counterstained (blue). **B**) F-actin arrangement in C8161-CON and C8161-ODAM cells stained with phalloidin as above in ‘**A**’.

In addition to effects on cell growth, adhesion, and motility, ODAM expression in MDA-MB-231 cells yielded cytoskeletal reorganization indicative of morphological reversion towards a more developed, epithelial phenotype, evident as increased vimentin solubility and F-actin rearrangement [18]. Cytoskeletal arrangement in control and ODAM-expressing melanoma cell lines was visualized by phalloidin staining and indicated clear morphologic changes associated with ODAM expression (Figure 2). The A375-ODAM cells exhibited smaller size compared to control cells, and an essentially complete disappearance of actin stress fibers, with a transition to circumferential actin cables. In addition, these cells adopted a more clustered arrangement in the cultures and showed a marked increase in formation of adherens junctions with localization of ß-catenin at cell-cell interfaces. In contrast to the A375-ODAM cells, C8161-ODAM cells adopted a larger, more rounded morphology relative to the spindle shape of cells in control cultures. These cells did not exhibit circumferential actin cables (Figure 2, bottom panel) or ß-catenin arrangement in adherens junctions.

### Analysis of signal transduction

**Figure 3:**
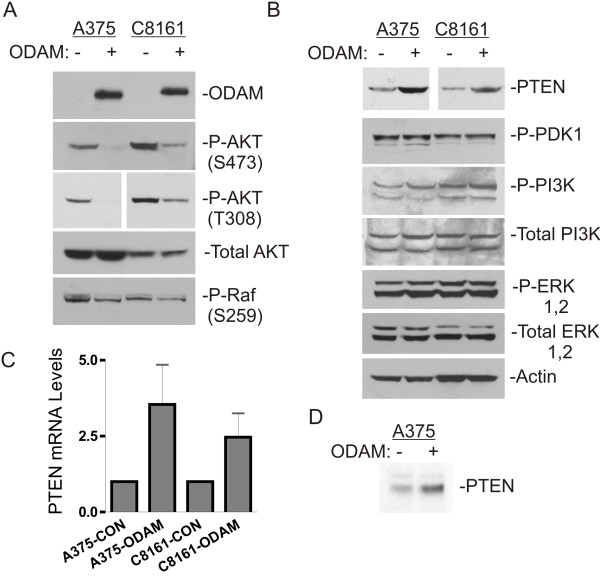
**Inhibition of AKT activation by ODAM expression in human melanoma cell lines. A,B**) Western blot analysis of AKT activation in total cell lysates from control and ODAM-expressing A375 and C8161 melanoma cells grown under normal culture conditions. Multiple blots from the same lysate sets were probed sequentially with the indicated antibodies. ODAM expression was detected by immunoprecipitation from cell culture supernatants **C**) Quantitative real time RT-PCR analysis of PTEN mRNA levels in control and ODAM-expressing cells growing under normal culture conditions. Values for ODAM-expressing cells represent the mean ± 1 S.D. from five independent determinations expressed relative to values from control cells assayed concurrently. **D**) Analysis of PTEN protein synthesis in control and ODAM-expressing A375 cells by metabolic labeling and immunoprecipitation as given in the methods.

Human melanomas frequently exhibit dysregulation of crucial signal transduction pathways and their components, including those of the Ras/Raf/MEK/MAPK and PI3K/AKT/mTOR pathways, each of which constitute central regulators of cell growth, survival, and other critical parameters of oncogenesis [6-9]. Western blot analysis of melanoma cell lysates with phospho-specific antibodies revealed a marked decrease in AKT activation in ODAM-expressing cells evident as decreased phosphorylation on both the Ser 473 and Thr 308 residues associated with AKT activation (Figure 3A), while overall levels of AKT protein were unaffected. Accordingly, phosphorylation of c-Raf (S259), a downstream target of AKT [24], was also decreased.

Activation of AKT requires the generation of phosphatidylinositol-3,4,5-triphosphate (PIP_3_) by phosphatidylinositol 3-kinase (PI3K), together with membrane docking of AKT and dual site phosphorylation of AKT by phosphoinositide-dependent kinase-1 (PDK1) and mTOR [25][26]. Conversely, activation of AKT is antagonized by the PTEN tumor suppressor gene product through its PIP3-phosphatase activity [27-29]. Probing of western blots with phospho-specific antibodies for active PDK1 and PI3K indicated no alterations in their activation state associated with ODAM expression (Figure 3B). Significantly, levels of PTEN protein were elevated (3–4 fold) in A375-ODAM cells relative to controls, and similarly in C8161-ODAM cells. Accordingly, measurements of PTEN mRNA by quantitative real time RT-PCR indicated that the PTEN message was increased (2.5-4 fold) in A375-ODAM and C8161-ODAM cells over those in vector control cells (Figure 3C). Metabolic labeling analysis confirmed the increased rate of synthesis of PTEN protein in A375-ODAM cells (Figure 3D).

In contrast to altered AKT activation, probing of blots with phospho-ERK 1 and 2 antibodies for active MAPK indicated that levels of phosphorylated (active) ERKs were no different in control and rODAM-expressing melanoma cells suggesting that signaling through this pathway is not directly altered by ODAM expression under these culture conditions (Figure 3B).

**Figure 4:**
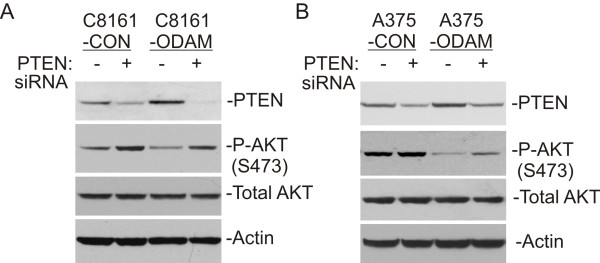
**AKT suppression by ODAM is PTEN dependent. A**) Western blot analysis of PTEN expression and AKT activation in whole cell lysates of C8161-CON and C8161-ODAM cells treated 72 hours with control or PTEN-specific siRNA as given in the methods. **B**) A375-ODAM and control cells were treated and analyzed for phospho-AKT and PTEN levels as in ‘**A**’.

Since PTEN is known to inhibit AKT activation we wished to establish whether the elevated PTEN levels evident in ODAM-expressing melanoma cells are responsible for the observed suppression of AKT activation. Therefore we treated cultures with control and PTEN-specific siRNAs and assayed PTEN levels and phospho-AKT by western blots of lysates prepared 72 hours later. As shown in Figure 4A, PTEN protein expression was substantially downregulated by specific siRNA treatment of both C8161-CON and C8161-ODAM cells and this corresponded with increased AKT phosphorylation in both cultures. While PTEN siRNA treatment reduced PTEN protein levels to a lesser degree in A375-ODAM cells, AKT phosphorylation was increased (Figure 4B).

**Figure 5:**
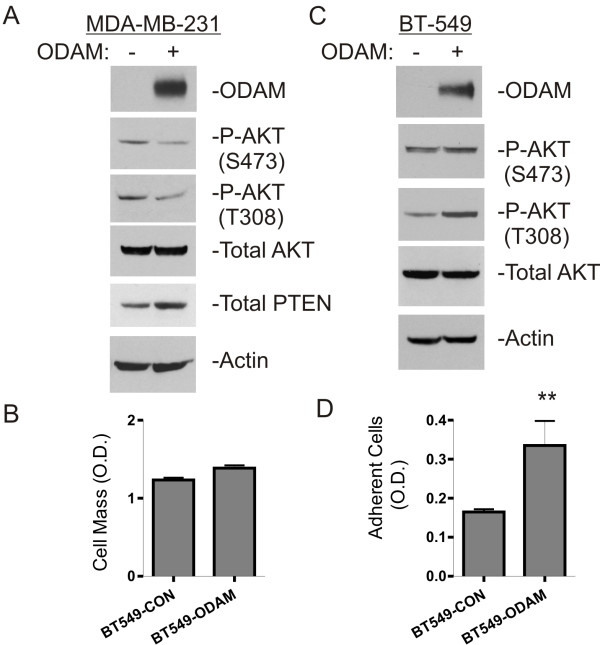
**ODAM inhibits AKT activation in MDA-MB-231 breast cancer cells but not in BT-549 breast cancer cells that lack PTEN expression. A**) Western blot analysis of AKT activation in lysates of control and ODAM-expressing MDA-MB-231 cells. Whole cell lysates were probed with the indicated antibodies as given in the methods. **B**) Growth assay of control and ODAM-expressing BT-549 breast cancer cells. Values represent the mean relative cell mass ± 1 S.D. from four replicate wells after 6 days in culture. **C**) Western blot analysis of AKT phosphorylation/activation in whole cell lysates of control and ODAM-expressing BT-549 cells. **D**) Matrigel adhesion assay of control and ODAM-expressing BT-549 cells (**, p< 0.01). Values represent the mean O.D. ± 1 S.D. for six replicates.

To test whether suppression of AKT activation and the elevation of PTEN expression is specific to ODAM-expressing melanoma cells or may be observed in other cell types, we examined AKT phosphorylation and PTEN expression in MDA-MB-231 breast cancer cells where we have also observed prominent anti-tumor effects upon ODAM transfection [18] Lysates of control and ODAM-expressing MDA-MB-231 cells were probed for phospho-AKT and PTEN expression and, as with the melanoma cell lines, MDA-MB-231-ODAM cells exhibited decreased AKT phosphorylation (2-fold) on the activating S473 and T308 residues and, correspondingly, 3-fold increased expression of PTEN protein (Figure 5A).

To further investigate the role of PTEN in AKT suppression by ODAM we utilized BT-549 breast cancer cells which are phenotypically similar to MDA-MB-231 cells but do not express functional PTEN [30]. Notably, BT-549 cells did not exhibit growth suppression in response to stable ODAM expression (Figure 5B) while Western blot analysis indicated that phospho-AKT levels are also unaffected by ODAM expression in these cells (Figure 5C), lending credence to the association of AKT suppression with increased PTEN and the observed growth inhibition in cells expressing ODAM. ODAM-transfected BT-549 cells do, however, show increased adhesion on Matrigel-coated plates indicating that ODAM expression in these cultures is functional in this respect and, further, that ODAM effects on cellular adhesion are to some degree independent of regulation through PTEN (Figure 5D).

## Discussion

ODAM protein expression has been demonstrated in a wide range of normal odontogenic, glandular, and epithelial renewal tissues [10-13] as well as in malignancies including odontogenic tumors, gastric cancer, breast cancer, lung cancer, and melanoma [14-16]. Prior retrospective studies of breast cancer patient biopsies indicated an increase in ODAM expression localized to the cell nucleus associated with advancing disease stage, yet this expression corresponded with improved survival for patients at each stage [17]. A recent study of melanoma patient specimens indicated that nuclear ODAM-expression correlates with sentinel lymph node metastasis in over 70% of cases, indicative of higher stage melanoma at diagnosis and poor prognosis requiring more aggressive therapeutic intervention [2,19]. These studies have left the role of ODAM in malignancy unclear since, in both breast cancer and melanoma, nuclear ODAM localization corresponds with advancing disease stage yet its influence on disease outcome seemingly differs.

With respect to cellular functions of ODAM, those indicated in ameloblasts are varied, and include an extracellular role at the cell-tooth interface in the junctional epithelium, roles in enamel maturation, and in the response to peridontal disruption [31,32]. ODAM is secreted [13,33] yet may also have a role in the cell nucleus regulating matrix metalloproteinase expression via direct chromatin binding [34]. ODAM has thus been suggested to be a matricellular protein exhibiting functions at cellular junctions, in cell signaling, and in direct gene activation [32]. Our previous studies indicated that ectopic ODAM expression in MDA-MB-231 breast cancer cells led to suppression of tumorigenic properties *in vitro* and in murine tumor models [18]. When the A375 and C8161 human melanoma cell lines were transfected with a gene construct encoding ODAM, their cellular properties were affected in a fashion similar to our studies in MDA-MB-231 cells. Specifically, their growth rate, and migratory ability was decreased and this was associated with increased cell matrix adhesion and morphologic/cytoskeletal rearrangement.

The most significant finding in our studies is the marked suppression of AKT phosphorylation/activation upon ectopic ODAM expression in both melanoma and breast cancer cell lines (Figures 3 and 5). Further, this inhibition of AKT activation was associated with elevated expression levels of PTEN protein, a negative regulator of AKT activation with an essential tumor suppressive role in multiple tissues [35-38]. Dysregulated, active PI3K/AKT/mTOR signaling promotes cell proliferation and survival, and is found in a wide range of tumor types, including melanoma [39]. PTEN expression is frequently absent or decreased in melanoma and many other cancers [40-43], with loss occurring through mutation, deletion, epigenetic silencing, and loss of heterozygocity [44,45]. The attendant activation of AKT, often in association with ß-catenin stabilization and MAPK activation, serves as a primary driver of growth and metastasis in these tumors [9].

Knockout mouse studies have demonstrated the tumor suppressive role of PTEN in multiple tissues, and indicate that PTEN function is gene-dosage dependent, as subtle changes in PTEN protein expression level yield significant functional consequences in terms of tumor growth and progression [46,47]. In each of the melanoma cell lines the increase in PTEN subsequent to ODAM expression was sufficient that AKT activation was profoundly inhibited, and was recovered upon specific silencing of PTEN expression (Figure 4). Accordingly, cell growth and AKT activity were unaffected by ODAM in BT-549 cells that lack PTEN.

As to the mechanism(s) of increased PTEN expression our studies indicate that this corresponds with increased levels of PTEN mRNA in ODAM expressing cells, and likely an increase in *de novo* protein synthesis (Figure 3). Regulation of PTEN expression is, however, highly complex, mediated at transcription in part by p53 [48]. Further, PTEN protein levels are regulated posttranslationally by ubiquitin-mediated proteasomal degradation elicited by the E3 ubiquitin ligase activities of NEDD4 (neural precursor cell expressed developmentally downregulated protein 4–1), XIAP (X-linked inhibitor of apoptosis protein), and others [49,50]. PTEN stability and function are further regulated through phosphorylation by casein kinase 2 (CK2), RhoA-associated kinase (RAK), GSK3ß and others [51-53], as well as by direct protein interactions with P-REX2a [54] and a host of other proteins [45,55]. Further studies addressing transcriptional regulation of the PTEN gene, PTEN protein stability, and function will be required to fully define the modes of PTEN regulation with respect to ODAM expression and effects on AKT activation.

In a parallel to our observations, overexpression of the matricellular protein SPARC (secreted protein acidic and rich in cysteine) inhibits growth [56] and migration [57] of MDA-MB-231 cells, and yields elevated PTEN and growth suppression in neuroblastoma cells [58]. SPARC is the ancestral gene of the SPARCL1 (SPARC-like 1 gene) which is, in turn, the putative progenitor of those in the secretory calcium phosphoprotein (SCPP) gene cluster on human chromosome 4 (at 4q 13.3) which includes ODAM, the α/ß and κ caseins, and FDC-SP (Follicular Dendritic Cell-Secretory Protein) [59,60]. Matricellular proteins can modulate tumor cell proliferation positively, or negatively, through a variety of mechanisms [61]. SPARC has been reported to function as a tumor suppressor in neuroblastoma, breast, pancreatic, lung and ovarian cancers, yet SPARC is associated with highly aggressive tumor phenotypes in melanomas and gliomas [62-64]. In notable similarity to ODAM action SPARC modulates cell-cell, and cell-matrix interactions, elicits cellular adhesive signaling, and exhibits differential nuclear localization dependent on cellular status [63,65,66].

In studies again similar to our observations, overexpression of the Profilin-1 actin-binding protein in MDA-MB-231 cells yields growth suppression and decreased tumorigenicity [67-69]. This is associated with inhibition of AKT activity dependent on elevated PTEN, and with altered cell motility, actin rearrangement, and increased formation of adherens junctions.

## Conclusions

Our studies demonstrate that ectopic ODAM expression in melanoma cell lines suppresses growth and migratory activity in these cells, while eliciting elevated PTEN expression and suppression of AKT activity. These observations are in agreement with the inhibition of tumorigenicity we previously observed in MDA-MB-231 breast cancer cells expressing ODAM [18]. This serves, however, to highlight the seemingly contrary association of ODAM expression with more advanced malignancies [17,19], and the need for clarification of the role(s) it may play in these tumors. This will hinge on further investigation into ODAM localization/functionality in the context of tumor cell variation. In this regard recent studies have shed light on the complex interactions between the PI3K/AKT/mTOR, Ras/RafMAPK, and/or Wnt/ß-catenin signaling pathways governing tumor growth and metastasis in melanoma, colon cancer, breast cancer, and others [9,70-72]. These interactions are proving determinative in terms of tumor behavior and are proposed to be predictive in terms of therapeutic responsiveness. Defining ODAM expression in relation to signaling pathways active across the range of tumor phenotypes will allow us to further clarify its role in tumorigenesis and delineate any relationship it may have to pathway-specific therapeutic intervention.

## Competing interests

The authors declare no financial or non-financial competing interests.

## Authors’ contributions

JSF participated in the study design, carried out cell assays, immunostaining, assays of signal transduction, and drafted the manuscript. LMF participated in study design, cell assays and immunostaining, and drafting of the manuscript. JEP carried out mRNA analysis, and participated in preparation of the manuscript. CTB participated in study conception and editing of the manuscript. JML and JLB participated in study conception and editing of the manuscript. AS participated in conception of the study, study design, and editing of the manuscript. DPK conceived of the study, and participated in its design and coordination and helped to draft the manuscript. All authors read and approved the final manuscript.

## Pre-publication history

The pre-publication history for this paper can be accessed here:

http://www.biomedcentral.com/1471-2407/13/227/prepub
